# Organizational and provider level factors in implementation of trauma-informed care after a city-wide training: an explanatory mixed methods assessment

**DOI:** 10.1186/s12913-017-2695-0

**Published:** 2017-11-21

**Authors:** April Joy Damian, Joseph Gallo, Philip Leaf, Tamar Mendelson

**Affiliations:** Johns Hopkins Bloomberg School of Public Health, Department of Mental Health, 624 North Broadway Street, 8th Floor, Baltimore, MD 21205 USA

**Keywords:** Trauma-informed care, Organizational culture, Secondary traumatic stress, Mixed methods

## Abstract

**Background:**

While there is increasing support for training youth-serving providers in trauma-informed care (TIC) as a means of addressing high prevalence of U.S. childhood trauma, we know little about the effects of TIC training on organizational culture and providers’ professional quality of life. This mixed-methods study evaluated changes in organizational- and provider-level factors following participation in a citywide TIC training.

**Methods:**

Government workers and nonprofit professionals (*N* = 90) who participated in a nine-month citywide TIC training completed a survey before and after the training to assess organizational culture and professional quality of life. Survey data were analyzed using multiple regression analyses. A subset of participants (*n* = 16) was interviewed using a semi-structured format, and themes related to organizational and provider factors were identified using qualitative methods.

**Results:**

Analysis of survey data indicated significant improvements in participants’ organizational culture and professional satisfaction at training completion. Participants’ perceptions of their own burnout and secondary traumatic stress also increased. Four themes emerged from analysis of the interview data, including “Implementation of more flexible, less-punitive policies towards clients,” “Adoption of trauma-informed workplace design,” “Heightened awareness of own traumatic stress and need for self-care,” and “Greater sense of camaraderie and empathy for colleagues.”

**Conclusion:**

Use of a mixed-methods approach provided a nuanced understanding of the impact of TIC training and suggested potential benefits of the training on organizational and provider-level factors associated with implementation of trauma-informed policies and practices. Future trainings should explicitly address organizational factors such as safety climate and morale, managerial support, teamwork climate and collaboration, and individual factors including providers’ compassion satisfaction, burnout, and secondary traumatic stress, to better support TIC implementation.

## Background

Traumatic experiences in childhood--often referred to as adverse childhood experiences (ACEs)--are common, particularly in low-income urban communities (HHS, 2014), with well-documented negative impacts on long-term development and functioning ([1]; Spitzer, 2007; [2]). There is growing support for trauma-informed care (TIC) trainings [3], which facilitate increased awareness of the needs of traumatized youth among youth-serving providers who have potential to serve as gatekeepers for trauma-informed services and resources. However, the impact of TIC trainings on contextual variables, including organization- and provider-level factors, is not well understood. As highlighted by implementation science theory, translation of evidence-based interventions into practice for addressing the needs of traumatized youth requires evaluation of contextual factors, including organizational climate and culture [4, 5] and the characteristics of providers [6, 7], since these factors influence adoption and sustainability of trauma-informed practices.

Implementation science methodologies hold great benefit for evaluating the real-world applications of the TIC framework. Implementation science is a growing field that rigorously investigates how best to translate research into public health practice [8]. Implementation science research suggests several organizational factors including *organizational culture*, e.g., a pattern of shared assumptions, attitudes, and beliefs in an organization ([9]; Damschroder, 2009; [10, 11]) and *organizational climate*, employees’ perceptions of management practices and procedures [12, 13], are important components of successful implementation of evidence-based interventions. More specifically, implementation studies have shown that organizations with cultures that are supportive of employees are more effective in implementing changes in the organization, including new interventions [14, 15].

Implementation science literature has also highlighted individual-level factors reflecting the implementation climate and the organization’s readiness for change. These factors include *change valence* [16], whether personnel think the change being implemented is personally beneficial or worthwhile, and *change efficacy* [16], the degree to which personnel think they are capable of implementing a change. Individuals’ willingness to adopt TIC policies and practices can be influenced by a number of organizational characteristics (i.e. culture and climate of an organization (Aarons, 2006); leadership (Aarons, 2007); level of organizational support for the intervention (Klein, 2001) and provider-level factors (i.e. attitudes and beliefs (Ajzen, 2005); self-efficacy and support (Bandura, 1982). Thus, it is critical to assess not only the intervention itself, but also the context in which the intervention is delivered.

Research on TIC interventions is emerging, but more comprehensive assessments of these interventions are needed. For example, some studies have evaluated knowledge about trauma and trauma-informed practices, including open-ended questions about lessons learned from trainings and their relevance to participants’ professional responsibilities (Anderson, 2015; Kramer, 2012; Nicola, 2013; Suzanne, 2016) without assessing contextual factors as direct outcomes. Other research assessed individual factors as outcomes without examining the effect of TIC trainings on organization-level outcomes (Layne, 2011; Keesler, 2016). To our knowledge, only three studies have assessed both organizational and provider level factors as outcomes [17–19]; however, these studies have solely focused on organizational and provider level factors within one sector e.g. child welfare only [17–19]. For example, Fraser et al. (2016) used the Trauma System Readiness Tool to assess participants’ perceptions of agency-level and personal knowledge and capacity to use of TIC practices. Although Hidalgo et al. [18] used a mixed methods approach and assessed quality of life of providers, the study was limited to residential care personnel. Research is needed that not only evaluates how TIC intervention informs service delivery to traumatized youth and families, but also how the intervention directly affects organizations and individuals providing those services across multiple service sectors.

The hospitalization and death of Freddie Gray, a 25-year-old Black American man in Baltimore in April 2015, resulted in a period of protesting against the reported mistreatment of Freddie Gray by the Baltimore Police Department. These events brought to national attention the chronic public health challenges of poverty, racism, and violence contributing to the high prevalence of trauma among youth and families in Baltimore. In response to the Freddie Gray tragedy and ensuing Baltimore unrest, the Baltimore City Health Department, together with its quasi-governmental partner Behavioral Health System Baltimore and support from the Baltimore City Office of the Mayor, developed the Healing Baltimore initiative through a grant from the U.S. Substance Abuse and Mental Health Services Administration (SAMHSA) Center for Mental Health Services, National Center for Trauma-Informed Care (NCTIC). The former Baltimore City Mayor Stephanie Rawlings-Blake pledged a commitment to have all frontline city workers trained in TIC. Thus, under the leadership of the Baltimore City Health Department, Baltimore City became the first city in the country to launch a citywide TIC training initiative for all government employees, which offered a unique opportunity for evaluation.

Our study had two main objectives. First, we evaluated agency personnel self-reported changes in organizational culture and provider-level compassion satisfaction and fatigue after completion of a nine-month TIC intervention that included agency implementation of TIC policies and practices. *Compassion satisfaction* is defined as the pleasure derived from being able to do one’s work well [20]. *Compassion fatigue* encompasses two components: *burnout*, which is associated with feelings of hopelessness and difficulties in being able to do one’s work effectively, and *secondary traumatic stress*, which involves developing problems due to exposure to the trauma of others [20]. Based on preliminary data from an earlier phase of the training and the intention of the training developers to address organizational and provider level factors associated with TIC implementation, we hypothesized that there would be a significant improvement in organizational culture, significant increase in compassion satisfaction, and significant decrease in compassion fatigue upon completion of the training.

Second, our study obtained participants’ perspectives on the training using individual in-depth interviews with a subset of participants. Quantitative studies on the organizational culture and providers’ professional quality of life employ established standards for measuring these domains. However, quantitative data may miss contextual detail regarding the impact of the training on participants, or how the training might be improved. Qualitative methods complement quantitative methods by providing detailed descriptions or narratives regarding the impact of the training, including trainees’ perceptions and experiences in participating in the intervention [21]. We used a sequential explanatory mixed methods design [22] in which open-ended interviews are used to provide explanation and context for survey responses. By applying a mixed methods approach to evaluating the training intervention, we aimed to reach a better understanding of participants’ experiences with the training and how the training could be improved [14, 23]. Thus, this study addressed gaps in the prior literature on the impact of TIC training by using a mixed methods design, including participants from multiple service sectors, and assessing organizational and provider factors as outcomes.

## Methods

### Healing Baltimore initiative training and sample

The Baltimore City Health Department (BCHD), in collaboration with SAMHSA’s National Center for Trauma Informed Care (NCTIC) and Behavioral Health System Baltimore (BCHB), led a nine-month comprehensive, evidence-based trauma-informed implementation training and coaching collaborative to agencies across Baltimore City. SAMHSA’s Concept of Trauma and Guidance for a Trauma-Informed Approach [3] provided the framework for this training intervention. NCTIC consultants conducted the monthly training at the BHSB office, and focused on educating and providing technical assistance to participants in implementing the six TIC principles outlined by SAMHSA: 1) Safety, 2) Trustworthiness and Transparency, 3) Peer Support, 4) Collaboration and Mutuality, 5) Empowerment, Voice and Choice, and 6) Cultural, Historical and Gender Issues.

Under this multi-systemic, multi-agency collaborative, government agencies and youth-serving organizations across Baltimore City participated in several activities including a series of monthly technical assistance, coaching, and feedback sessions from national trauma experts on how to utilize trauma-informed practices at their agency. The monthly implementation training sessions were co-facilitated by the same two trainers throughout the intervention. Participants represented a wide range of government agencies and nonprofit organizations that interact with traumatized persons. Participating agencies can be categorized as falling within the following domains: Law Enforcement, Social Services, and Health and Education. Participants in the nine-month training (*N* = 90) were identified by their respective agencies to lead and implement trauma-informed approaches at their respective workplaces. No predetermined, uniform selection criteria were used across all agencies; rather, selection of participants was at the independent discretion of the participating agencies. All participants were over 18 years of age and English speaking. The Institutional Review Board at the Johns Hopkins Bloomberg School of Public Health approved all study procedures, and all research participants provided informed consent.

### Pre-post design

Data came from pre- and post-surveys administered by BCHD and BHSB staff at the beginning (October 2015) and at the conclusion (June 2016) of the nine-month implementation training. The pre- and post-surveys were administered to all training participants (*N* = 90). An online version of the survey was administered to participants not present at either the first or last training of the intervention. Participants were compensated for their time by their respective agencies.

### Measurement strategy

This study employed a subset of measures from the pre-post surveys regarding changes in provider-and organizational-level factors associated with implementation of trauma-informed care, as described below.

*Safety Attitudes Questionnaire (SAQ).* The SAQ is a validated 60-item questionnaire which measures organizational factors such as safety climate and morale, work environment factors such as managerial support, and team factors such as teamwork climate and collaboration [24]. Participants responded to all items using a Likert scale ranging from 1 *(disagree strongly)* to 5 *(agree strongly).* The scale demonstrated an acceptable level of internal consistency in our study sample (Cronbach’s alpha = .77).

*Professional Quality of Life (ProQoL).* The ProQoL scale is a validated 30-item self-report assessment of work-related 1) compassion satisfaction, 2) burnout, and 3) secondary traumatic stress [20]. Participants responded to all items using a Likert scale ranging from 1 *(never)* to 5 *(very often).* Both subscales demonstrated strong internal consistency in our sample: Cronbach’s alpha = .88 for compassion satisfaction and .87 for compassion fatigue.

*Other covariates*. BCHD staff also administered a brief form to gather information about sociodemographic characteristics such as organization affiliation (government law enforcement, government social services, government health education and nonprofit), race/ethnicity (African-American, White, Latino, Asian or Pacific Islander, Other), gender, age (18–34, 35–44, 45–54, ≥ 55), educational attainment (high school, some college, college, graduate degree), role at agency/organization (direct service, management/administration), years in current position (< 1 year,1–5 years, 6–10 years, 11+ years), native of Baltimore City (yes/no), and participation in any prior TIC training (yes/no).

### Semi-structured interviews

Semi-structured interviews were conducted two months following the intervention with a subset of participants (*n* = 16) in the intervention. BCHD and BHSB staff overseeing the TIC intervention described the study to all trainees, asked them if they were interested in learning more about the study, and shared the contact information of interested trainees with the research team. Snowball sampling was used to recruit additional participants for the interviews; initial interviewees were asked to recommend others from their respective agencies who might be interested in participating in the training. The lead author explained the study and obtained informed consent. Interviews were digitally recorded and transcribed. Any identifying information, such as the names of individuals or places, was removed in the transcription process.

The interview guide for semi-structured interviews covered four domains based on discussions with key informants (i.e. BCHD staff, SAMHSA training developers) and preliminary research (Damian, 2015): 1) Usefulness of training, 2) General impact of training on organizational culture and climate, 3) Specific impact of training on organizational culture of safety, and 4) Impact of training on referrals of traumatized individuals. In this study, we focused on responses pertinent to the second and third domains listed above (i.e., impact of training on organizational culture and climate and organizational culture of safety). The first author conducted all interviews.

### Analysis strategy

Data were first inspected for data entry errors and outliers and cleaned as needed. Multiple imputation via chained equations (van Burren, 1999) was applied to missing data (>5% of values were missing). Paired t-tests were conducted to examine if mean SAQ and PROQoL scores changed significantly from the beginning to the conclusion of the 9-month intervention training. Multiple regression analysis was employed to adjust for the potential confounding effects of the demographic variables noted earlier in this paper on the relationship between pre-post SAQ and PROQoL mean scores. Wilk’s test was used to test the assumption of normality. STATA 13.0 was used for all statistical analyses.

Analysis of semi-structured interviews was conducted by two trained coders who independently used the constant comparative method, moving iteratively beyond codes and text to derive themes related to what participants thought about the intervention. Originally developed for use in the grounded theory method of Glaser and Strauss [25], the constant comparative method involves selecting one component from the data, such as a theme, and comparing it to the rest of the data to develop conceptualizations about possible relations across various data components [26]. We focused our attention on responses related to how the intervention influenced participants’ organizational culture and climate with an additional interest in the impact on organizational safety.

## Results

### Sample characteristics

Of the 90 pre-survey respondents, we excluded 2 (2.2%) who only completed the sociodemographics questions but did not respond to the rest of the survey. Table 1 shows the demographics of the analytic sample (*N* = 88). The mean age of study participants was 43.0 years (standard deviation: 13.6), and most were women (74.1%) and African American (71.4%). Most participants had at least a college degree (77.0%) and had previously participated in some form of trauma-informed care training (60.0%). Sixteen participants, mostly women (87.5%), volunteered for interviews and were individually interviewed by the first author.

**Table 1:** Demographics of Analytical Sample in Baltimore City Health Department’s Trauma-Informed Care Training Intervention (*N* = 88)

Characteristics	Total^a^ N (%)
Age, in years	
Less than 35	26 (44.8)
35–44	14 (24.1)
45–54	10 (17.4)
55 or more	8 (13.8)
Gender	
Male	22 (25.3)
Female	65 (74.1)
Race/Ethnicity	
African-American or Black	60 (71.4)
White	21 (25.0)
Latino	1 (1.2)
Asian or Pacific Islander	1 (1.2)
Other	1 (1.2)
Highest level of education completed	
High school	5 (6.76)
Some college	12 (16.2)
College	20 (27.0)
Graduate degree	37 (50.0)
Role at agency/organization	
Direct service (Frontline)	39 (44.3)
Management/Administration	49 (55.7)
Years in current position	
Less than a year	18 (20.7)
1–5 years	37 (42.5)
6–10 years	14 (16.1)
11+ years	18 (20.7)
Baltimore City native	
Yes	37 (43.0)
No	49 (57.0)
Participated in any prior trauma-informed care training	
Yes	51 (60.0)
No	34 (40.0)

^a^Column percentages may not add up to 100% due to missing values

(Data from pre-post surveys administered during Baltimore City Health Department’s TIC Intervention)

### Change in organizational culture and climate

The improvement in mean SAQ scores (M = 3.91, SD = 17.04, β = 0.58; *p* < 0.05) from pre- to post-training was significant (Table 2). Additionally, mean scores for the compassion fatigue (M = 11.76, SD = 20.16 β = 1.00; *p* < 0.001) and compassion satisfaction (M = 7.97, SD = 10.91, β = 1.00; p < 0.001) subscales of the PROQoL scale both increased significantly.

**Table 2:** Pairwise Comparisons of Safety Attitudes Questionnaire (SAQ) and Professional Quality of Life (PROQoL)

	Baseline Mean (SD)	Post Mean (SD)	Pre-post Difference (SD)	Effect Size	95% CI for Difference	
SAQ	78.97 (11.51)	82.88 (12.19)	3.91 (17.04)*	−0.16	0.30	7.52
PROQoL						
Compassion Fatigue	43.09 (9.77)	54.85 (17.07)	11.76 (20.16)***	−0.39	7.49	16.03
Compassion Satisfaction	39.73 (6.12)	31.76 (8.74)	7.97 (10.91)***	0.47	5.65	10.28

Increased *Compassion Fatigue* and *Compassion Satisfaction* scores mean higher level of compassion fatigue and satisfaction, respectively

*p < 0.05 ***p < 0.001

(Data from pre-post surveys administered during Baltimore City Health Department’s TIC Intervention)

### What participants said about the training

Four major themes relevant to the SAMHSA TIC principles outlined earlier in this paper emerged from our review of the qualitative data (Table 3), two related to changes in organizational-level factors and two related to changes in provider-level factors.

**Table 3:** Themes of intervention usefulness identified through qualitative analysis of participant interviews *(n = 16)*

Theme	Sample quotes
Implement more flexible, less-punitive policies towards clients	*We like to think that the way we implement it (programs) is very compassionate and trauma-informed. One of the things that we definitely have shifted since doing the training is that we aren’t quite as rigid. We’ve loosened up a bit. We’ve become less attached to how it has to look, as far as the session goes. We’re meeting them more where they are.* [social services #002]
	*If somebody comes down and were having a difficult conversation, we don’t force the session, we allow clients to retreat, we check on them later…and if a client needs a break we say how about we pick up this conversation later. If somebody is extremely agitated, we sit in silence and help them catch their breath, we try and maintain a steady tone of voice with them…we talk about ways that they can take care of themselves between that session and the next session.* [social services #008]
	*Just more patience. More understanding, and to not write a kid off as – as being inherently bad or “young criminal” or something. You kind of understand that there are different things at play.* [law enforcement #001]
	*And the other component that has helped me is not making it so rigid... I sometimes have people who seem nervous, so I sit with them outside and we just sit outside and talk. And I don’t need to have the papers in front of me and have notes. I can just go back and write that we had a meeting and write it down and it helps them not be so nervous to talk to me. Those are the things that have been really helpful to keep in the back of my mind to remember that people may not be comfortable in this room, they may not be comfortable talking to me for lots of reason. Those have been really helpful.* [law enforcement #002]
	*For me I made stuffed animals available for the children. I switched the way I talked and how I approach them. Knowing that the way that the child is used to an authority figure talking to them is always yelling. So lowering my voice and changing the inflection in my voice. I offer them hugs. I ask them what do you need today to be successful.* [parks and rec #001]
	*Just a better, better sense of listening. I let them vent, I let them scream, I let them howl, I let them yell. You know whatever it takes for them, and when you’re quiet then the person says, “Hello, you still there?” “Yes ma’am, I’m still here, I’m just listening to you.” You know, so that I know that our house has served you, how to get you to the right person if I can’t help you, you know.* [311 operator #001]
Adopt trauma-informed workplace design	*We have set up our offices a little differently, we give them options to either face the door or have their back to the door. we have redone our front office so it’s a little less chaotic and a little more soothing when they come in.* [social services # 003]
	*I recognize that you can actually design workspace in the environment in a remarkable easy and cost effective way to implement the trauma informed approach to care* [social services # 004]
	*We all discussed it and we felt like clients when they came into the office were not feeling welcomed and that had a lot to do with the pictures on the wall, it had a lot to do with the color of the office, and so we had volunteers come in and they had redesigned our office and they painted so it’s more brighter, it’s more calming, you know, the pastel colors, as opposed to dark or gray or like, hospital or padded entry (?) type, one color wall. We’ve made it more brighter, like I said, we changed the layout of the desks. They’re now, they’re going to be in an L-shape as opposed to when you come in, there’s an I.* [social services # 005]
	*You make sure that their space is clean and that it smells and good and you know so that’s the kind of physical and environmental stuff that I integrate into the work. I turned off the light in my office and put up a lamp in it to make it a softer light… I had brought stuff for children in my office. I had done that before, but stopped it. I – I brought it back in. I have kind of adjusted the arrangement of my desk and how the residents approach my door, I notice a whole difference in my interactions with them that does not lead to me being overly stressed out.* [social services #007]
Heightened awareness of own traumatic stress and need for self-care	*The training really prompted me to look at my own trauma and figure out if I was really able to access it. I’d say I’ve become more inclined, like, to be aware of – like, “Hey, you know, I’m kinda stressed. I need to take some time off.” I definitely realize that I’ve got stuff going on, that whole secondary trauma thing. I’m more aware of that than I was before. The need to do that. It’s very hard for me because, you know, I’m kind of 24/7. I have my phone on all the time… I have always understood self- care and I you know, I have started writing, or I started painting, or I dance, or I journal… I think the training started to bring me back to a place where I needed to do some introspection. Introspection I needed to find an outlet, to really take care of myself.* [social services #001]
	*Now I take more time for myself. I’ve learned not to feel guilty, not thinking about the residents when I get home. I started it about two years ago- I have two corners that I turn to get home. Once I turn that second corner, I had to train myself to let go and I will be back tomorrow because that’s when I have to start focusing on me, my children, and my home, I know I did a good job, so while I’m driving those two corners, I think about what good I did in a day and what did I accomplish and I give myself a pat on the back, and I keep it moving. So that’s how I train myself not to bring the job home*. [social services #005]
	*After our training was completed, we had a pretty in-depth team meeting around self-care. We do encourage especially more aggressively taking those mental health days. I make it a priority to try and kind of des-stress and create an environment for myself at home, not bringing work home… I do my best, but you know sometimes it’s just a really crappy day and you can’t just say I’m just gonna leave that at work and not bring that home*. [social services #009]
	*You’re feeling a certain way and you don’t really understand it and then you get to subjective presentation of the effects of secondary trauma and, um, how that affects you and you’re like, “Oh, okay. That’s kind of why I feel the way I do.’* [law enforcement #001]
Greater sense of camaraderie and empathy for colleagues	*When you’re not at a good place or feeling overwhelmed by the work, or feeling stuck, there is a willingness and openness on supervisors that were aware of it, and were able to support staff in a way that’s not making them feel like they’re incompetent or that they are failing.* [social services #001]
	*Senior management keeps an open-door policy where we can um come to them if there, you know, is anything bothering us or anything that we just need to get off our chest, anything anytime we need to express any concerns, um that they definitely maintain an open-door policy for us. I do the same for my stuff um and we make sure that we have certain um procedures in line so that um if someone does start to feel um burnt out that they, they have that access to um have that, take the time off that they need or you know take that break that they need, so that we can encourage that, that healthier workplace*. [social services #008]
	*I do the same for my staff and we make sure that we have certain procedures in line so that if someone does start to feel burnt out that they can take the time off that they need or take that break that they need, so that we can encourage that, that healthier workplace.* [social services #009]
	*I feel like you helped me understand my fellow officers better. And then, um, I saw, even though I’m not patrol anymore, I saw how understanding trauma-informed care would help, you know, patrol officers, um, kinda go into a situation with a little bit more confidence and understanding.* [law enforcement #001]
	*I try to recognize that the secondary trauma and the burnout from the things that we do so I try to make sure that the staff have breaks that they need, if I just tag team if you need to tag out and get a little breather I can tag in or we can rotate the groups so that it’s possible that they can kind of “woo-saw” and take that deep breath and then we have staff meetings regularly, kind of changed the format to allow more open discussion instead of, what happens is the center directors have meetings with our area managers and so instead of it just being information being disseminated to kind of do pulse checks with the staff.* [Parks and Rec #001]
	*We had several cookouts, well cook-ins, several cook-ins. We had a relaxed dress code for probably about a month and a half; we’re still in it till September 2nd. That has kinda lifted everybody’s spirit a little bit. Try to bring everybody together as a whole because we as a group, we were really divided, so it’s kind of brought some of us together.* [311 operator #001]

Theme 1: Implement more flexible, less-punitive policies towards clients n = 9 (56%)

Theme 2: Adopt trauma-informed workplace design *n* = 4 (25%)

Theme 3: Heightened awareness of own traumatic stress and need for self-care *n* = 10 (63%)

Theme 4: Greater sense of camaraderie and empathy for colleagues *n* = 11 (69%)

*Themes related to changes in organizational-level factors.* Two themes highlighted organizational changes that took place to improve trauma-informed practices with youth and families using services. Nine out of 16 participants reported agencies’ implementation of more flexible, less punitive policies towards clients. Participants explained that policies and procedures at their respective organizations were changed to meet the real-time needs of clients. Participants also observed greater organizational awareness of the need to prioritize empathy and meeting clients where they are at, as opposed to being solely focused on completing paperwork. The second theme that emerged related to organizational change was adoption of a trauma-informed workplace design (reported by 4 out of 16 participants). Participants described how their respective agencies adjusted the physical layout of their workplace to be more welcoming and to serve as a calm, safe space for clients. They reported that a change in the physical space facilitated more positive interactions between them and their clients.

*Themes related to changes in provider-level factors.* Two additional themes highlighted the impact of the intervention on changes at the provider level. Eleven out of 16 participants reported a greater sense of camaraderie and empathy for colleagues. This theme was consistent with survey results regarding participants’ improved compassion satisfaction score; participants reported greater initiative on the part of senior management at their respective organizations to appreciate providers’ efforts, as well as enact policies to enhance providers’ well-being. A second provider-level theme was a heightened awareness of providers’ own traumatic stress and need for self-care (reported by 10 out of 16 participants). Participants’ description of becoming more aware of their own trauma and traumatic stress were consistent with the survey findings of participants’ increased compassion fatigue. Participants explained their need to set boundaries at work and practice healthy ways of releasing stress experienced from interacting with traumatized individuals.

## Discussion

Our study highlights significant changes that occurred among government and nonprofit service personnel representing a range of sectors (Health and Education, Law Enforcement, Social Services) who participated in a nine-month TIC implementation training and learning collaborative. The findings suggest that research participants generally perceived the TIC intervention as beneficial for themselves and their organizations. With respect to program benefits, the training intervention showed positive impacts both with respect to organizational factors (safety climate and morale, managerial support, teamwork climate and collaboration) and individual factors (compassion satisfaction). Although the intervention in this study uses a different training model and framework for understanding TIC, the findings in this paper are consistent with previous studies [18], which also found improvement in safety climate and job satisfaction among residential care providers that participated in a TIC training. Implementation studies have shown that organizations with cultures that are more supportive of employees are in turn, more effective in implementing changes in the organization ([14]; Klein, 2001). Thus, the observed improvements in organizational- and provider-level outcomes have potential to support participating agencies in better addressing the needs of trauma-affected youth [4, 10].

Our sequential, mixed methods design allowed us to use information derived from the semi-structured interviews to support the findings from the pre-post surveys and to point the way toward improved training. For instance, qualitative findings were largely consistent with survey results regarding TIC training impact on organizational-level factors. Participants’ reports of adoption of a trauma-informed workplace design supported significant quantitative findings regarding improvements in safety climate and working conditions. Some organizations restructured the physical layout of their offices as a demonstration of their commitment to providing a physically and psychologically safe space for clients and staff. Importantly, change in the physical working conditions was reported to enhance positive interactions between clients and staff.

Our findings also suggested significant changes in provider-level perceptions. Survey data indicated an overall improvement in perception of the quality of the work environment and approval for managerial action to promote a culture of safety. Our quantitative findings are consistent with the theme that emerged in individual interviews of a greater sense of camaraderie and empathy for colleagues. Some participants reported greater initiative on the part of management to support staff through open door policies and establishing mental health days, which is consistent with our quantitative findings of improved perceptions of management. Our findings are in contrast to Lang and colleagues [19] who found that participants perceived low agency support for addressing trauma among children and staff experiences of secondary traumatic stress. Hidalgo et al. [18] found significant improvement in staff collaboration, but did not explicitly discuss changes in management support.

Our findings also highlighted providers’ increased awareness of their own personal stress. The survey items asked about feeling overwhelmed, worn out, and having difficulty in separating one’s personal life from one’s role as a provider. Participants’ interview responses reinforced quantitative findings regarding negative effects on job performance of secondary exposure to traumatically stressful events, and were consistent with previous studies (Fraser, 2016; [18, 19]), highlighting the high rate of secondary traumatic stress and burnout among service providers working with traumatized youth. The intervention likely shifted participants’ awareness of their emotional needs and stressors rather than increasing those stressors, which may be an important step for providers in moving toward better self-knowledge and self care. In addition to acknowledging their personal secondary traumatic stress, participants also reported greater awareness that their colleagues were experiencing a similar phenomenon, which subsequently increased staff empathy towards one another.

Qualitative data suggested that TIC training resulted in more flexible, less punitive policies towards clients; this was a unique finding captured by the semi-structured interviews. While the quantitative assessments provided information regarding changes in organizational and provider factors associated with implementation, the qualitative theme regarding policy changes revealed a direct impact of training on TIC implementation. Across sectors, a subset of interview participants described being less inclined to use labels and less rigid in their approaches to clients. Rather, these providers described being able to listen more and pay more attention to what the client needs at a given time. This is consistent with findings from semi-structured interviews conducted by Hidalgo et al. [18], in which participants also described reducing the use of restraints and improving communications with traumatized youth.

*Directions for Future Research, Practice, and Policy.* As noted earlier in this paper, previous studies on TIC trainings have largely included participants from the social services and healthcare sectors. However, as a recent synthesis of the literature on TIC pointed out [27], there is variation in the duration and level of engagement between providers and traumatized individuals based on providers’ employment sector, and these differences may influence implementation. Thus, future research should assess provider-client relationship dynamics as they relate to implementation, particularly in sectors where TIC training has been understudied, including law enforcement. Additionally, there is also a need for more studies on the efficacy of TIC trainings and changes to organizational practice [27]. Although the current study found significant organizational and provider level TIC changes following completion of training, there is a need to examine whether early adopters of TIC sustain the reported changes. Last, the findings underscore the high prevalence of secondary traumatic stress and burnout among service personnel that work with traumatized youth. Although there is a body of literature on trauma and secondary traumatic stress among providers and how these provider-level factors negatively effect job performance and quality of care provided to clients ([28–30]; Salters, 2016), future research is needed to evaluate how participation in a TIC training intervention helps providers deal with their own trauma, and the subsequent effects on quality of care provided to traumatized youth and families.

Although participants in the current study were involved in a fairly long (nine-month) training, the implementation literature on TIC [31] suggests the need to address system-level factors related to maintenance of intervention practices, particularly booster sessions, ongoing support/supervision and technical assistance to facilitate implementation. Thus, developers of the TIC training should consider the ways in which organizations and individual providers can continue to be supported beyond their formal participation in the training. In addition, given the significant increase in compassion fatigue suggesting a heightened awareness of providers’ burnout and secondary traumatic stress, future iterations of the training can include a more direct focus on providers’ mental health issues, including addressing potential stigma around mental health.

While this training largely focused on organizations and providers, it is important to recognize that traumatized youth are part of the TIC ecological model. Thus, as highlighted by Kramer & Burns [31], it is important to assess implementation culture at the level of the client and ensure that TIC trainings that educate youth and families about trauma are also made available. The framework set forth by Raghavan et al. [32] regarding implementation of evidence-based practices in public mental health settings, can also be applied to understand the policy implications of this study. Although this study focused on direct changes to organizational and provider factors resulting from participation in the training, the burden of implementation cannot be placed on any individual organization or provider. There is a need for development of policy, such as states creating a rewards structure for TIC, to support organizational and provider implementation. Moreover, given the time and resources required for employees to attend trainings and change organizational practices to be more trauma-informed, policies can further incentivize implementation of TIC through reimbursement strategies and allocation of CEU professional credits by licensing boards.

*Limitations and strengths.* This study has several limitations. First, the lack of a comparison group limits the ability to estimate intervention effects. Recruitment of a comparison group for this study was not feasible since all City agencies participated in the TIC initiative and identifying comparison individuals at the participants’ respective agencies was beyond our funding and timeline constraints. Second, the participants in the intervention were recruited by their respective agencies with no uniform, predetermined selection criteria to select participants; therefore, their responses and any observed changes may not be representative of the other personnel at those agencies. Third, the participants in our semi-structured interviews may also not have been representative of the intervention participants. Fourth, the study relied on participants’ self-report, which is subject to socially desirable responding. Fifth, the study was underpowered to detect and compare differences in treatment effects across the different service sectors represented in the current study. Although an item on participants’ agency/organizational affiliation was included in the survey, only 25% of respondents answered this item. Consequently, we could not link or compare changes by agency/organization. Lack of information about fidelity to training and number of sessions attended is also a limitation. Lastly, the observed increase in providers’ compassion fatigue may result from factors external to the TIC training rather than from increased awareness. For instance, the finding may reflect an actual increase in trauma among providers’ clients or workload involving traumatized youth and families, which was not measured in this study.

This study also has a number of strengths. Use of a mixed-methods approach provided a nuanced understanding of the impact of the TIC training. The purpose of the open-ended interviews was exploratory, striving for depth of understanding and not representativeness. The current study was also strengthened by the inclusion of diverse participants, with respect not only to race/ethnicity, gender, and role at their respective organization but also the various types of government agencies and nonprofit organizations they represented. The cross-sector representation of diverse agencies reflects the multiple service systems that come into contact with traumatized youth.

## Conclusion

Implementation science research indicates that both organizational and provider-level factors are important components of successful implementation of interventions such as TIC. Restructuring of TIC trainings to address organizational factors such as safety climate and morale, managerial support, teamwork climate and collaboration, and individual factors including providers’ compassion satisfaction, burnout, and secondary traumatic stress, can potentially support successful implementation of TIC policies and practices. In doing so, TIC trainings go beyond increasing participants’ knowledge about the biological and psychosocial consequences of trauma to incorporate the contextual (organizational and individual) factors associated with TIC service delivery. Attention to organizational and individual factors might enhance implementation of TIC principles. Expansion of cross-sector TIC trainings and evaluation of subsequent implementation of TIC related changes can help break down silos between different service systems and foster improvements in addressing the unique needs of youth that have experienced trauma. As the need for TIC receives greater attention and more resources are allocated to train personnel outside the traditional healthcare system in TIC, additional evaluation studies should be conducted to test long-term changes in participating organizations and individual providers.

## Abbreviations

ACEs: adverse childhood experiences
BCHB: Behavioral Health System Baltimore
BCHD: Baltimore City Health Department
NCTIC: National Center for Trauma-Informed Care
ProQoL: Professional Quality of Life
SAMHSA: Substance Abuse and Mental Health Services Administration
SAQ: Safety Attitudes Questionnaire
TIC: trauma-informed care
